# Machine learning for high-throughput field phenotyping and image processing provides insight into the association of above and below-ground traits in cassava (*Manihot esculenta* Crantz)

**DOI:** 10.1186/s13007-020-00625-1

**Published:** 2020-06-14

**Authors:** Michael Gomez Selvaraj, Manuel Valderrama, Diego Guzman, Milton Valencia, Henry Ruiz, Animesh Acharjee

**Affiliations:** 1 International Center for Tropical Agriculture (CIAT), A.A. 6713 Cali, Colombia; 2 Department of Soil and Crop Sciences, Texas A&M University, College Station, TX USA; 3 College of Medical and Dental Sciences, Institute of Cancer and Genomic Sciences, Centre for Computational Biology, University of Birmingham, Birmingham, B15 2TT UK; 4 Institute of Translational Medicine, University Hospitals Birmingham NHS Foundation Trust, Birmingham, B15 2TT UK; 5 NIHR Surgical Reconstruction and Microbiology Research Centre, University Hospital Birmingham, Birmingham, B15 2WB UK

**Keywords:** Automated aerial image processing, Above-ground biomass, Cassava, Machine learning, Multispectral UAV imagery, Root yield prediction

## Abstract

**Background:**

Rapid non-destructive measurements to predict cassava root yield over the full growing season through large numbers of germplasm and multiple environments is a huge challenge in Cassava breeding programs. As opposed to waiting until the harvest season, multispectral imagery using unmanned aerial vehicles (UAV) are capable of measuring the canopy metrics and vegetation indices (VIs) traits at different time points of the growth cycle. This resourceful time series aerial image processing with appropriate analytical framework is very important for the automatic extraction of phenotypic features from the image data. Many studies have demonstrated the usefulness of advanced remote sensing technologies coupled with machine learning (ML) approaches for accurate prediction of valuable crop traits. Until now, Cassava has received little to no attention in aerial image-based phenotyping and ML model testing.

**Results:**

To accelerate image processing, an automated image-analysis framework called CIAT Pheno-i was developed to extract plot level vegetation indices/canopy metrics. Multiple linear regression models were constructed at different key growth stages of cassava, using ground-truth data and vegetation indices obtained from a multispectral sensor. Henceforth, the spectral indices/features were combined to develop models and predict cassava root yield using different Machine learning techniques. Our results showed that (1) Developed CIAT pheno-i image analysis framework was found to be easier and more rapid than manual methods. (2) The correlation analysis of four phenological stages of cassava revealed that elongation (EL) and late bulking (LBK) were the most useful stages to estimate above-ground biomass (AGB), below-ground biomass (BGB) and canopy height (CH). (3) The multi-temporal analysis revealed that cumulative image feature information of EL + early bulky (EBK) stages showed a higher significant correlation (*r* = 0.77) for Green Normalized Difference Vegetation indices (GNDVI) with BGB than individual time points. Canopy height measured on the ground correlated well with UAV (CHuav)-based measurements (*r* = 0.92) at late bulking (LBK) stage. Among different image features, normalized difference red edge index (NDRE) data were found to be consistently highly correlated (*r* = 0.65 to 0.84) with AGB at LBK stage. (4) Among the four ML algorithms used in this study, k-Nearest Neighbours (kNN), Random Forest (RF) and Support Vector Machine (SVM) showed the best performance for root yield prediction with the highest accuracy of R^2^ = 0.67, 0.66 and 0.64, respectively.

**Conclusion:**

UAV platforms, time series image acquisition, automated image analytical framework (CIAT Pheno-i), and key vegetation indices (VIs) to estimate phenotyping traits and root yield described in this work have great potential for use as a selection tool in the modern cassava breeding programs around the world to accelerate germplasm and varietal selection. The image analysis software (CIAT Pheno-i) developed from this study can be widely applicable to any other crop to extract phenotypic information rapidly.

## Background

Cassava (*Manihot esculenta* Crantz), commonly referred as manioc (French), yuca (Spanish), and different names in local regions, is a tropical root crop native to South America [1], and relied by more than 800 million people as a staple food source [2]. Its versatile nature, it is often referred to as the “drought, war and famine crop of the developing world” [3], places it among the most adaptive crops during climate change. Early vigor, rapid root bulking, higher root yield, resistance to major pest and diseases, waxy cassava are the most important targeted traits in cassava breeding programs around the world [4]. Conventional breeding continues to be the main method for cassava varietal development worldwide and had a strong impact on addressing the constraints of cassava growers [5]. Traditional methods of selecting breeding/germplasm lines are labor intensive and destructive to nature, limiting the quantitative and repeated assessments in long-term research [6, 7]. Therefore, establishing a non-destructive and real time monitoring tool to measure above and below-ground cassava traits are very necessary [8]. Exploring non–destructive selection methods has always been a priority in cassava breeding programs. Therefore, efforts have been taken to reduce the cassava selection cycle and develop non-destructive, low-cost phenotyping methods that precisely measure the root characteristics in the field [8–12]. Though good progress in digital phenotyping has been made, so far, no studies have been devoted to the development of non-invasive high-throughput field phenotyping (HTFP) tools and machine learning models that estimate cassava canopy traits and root yield prediction through aerial imaging. In cassava breeding programs, the establishment of non-destructive phenotyping tools, root yield prediction models can allow the early selection of elite genotypes, allowing the optimization of resources and time [13]. Digital and rapid phenotyping approaches are increasingly considered important tools for rapid advancement of genetic gain in breeding programs [14].

UAV are being used to measure with high spatial and temporal resolution capable of generating useful information for plant breeding tasks [15–17]. In the era of digital revolution, aerial image phenotyping [18–20] and ML models could predict crop yield performance [21–27] in a non-invasive means with a greater accuracy [28–31]. Efficient selection of desired phenotypes through HTP across large field populations could be achieved through incorporating ML methodologies such as, automated identification, classification, quantification and prediction [20]. To be constructive to breeding programs, phenotyping methods must be robust, automated, sensitive, and amenable to plot sizes. The ability to get more rapid growth responses of genetically different plants in the field and transmit these responses to individual genes, novel technologies such as proximal sensing, robotics, integrated computational algorithms and robust automated aerial image analytical frameworks are urgently needed [7].

Even though, UAV and sensor technologies (hardware) shows greater progress with more automation and integration, processing the massive amount of generated image data such as data management, image analysis, and result visualization of large-scale phenotypic data sets [32] from aerial phenotyping systems requires robust analytical framework for data interpretation [33]. Few commercial software are available that systematize image calibration and correction, obtaining good field maps of the studied variable. But these platforms are often developed and delivered by specific enterprises where the original hardware and software are patent protected and henceforth cannot be adapted or modified to meet particular research needs [34]. Moreover, new developments target real-time processing on-board in aerial imaging platforms, providing direct vegetation indices (VIs) maps to make rapid decisions [35]. Despite these improvements, there are middle steps that require some level of manual interface, which slow the progress, such as the recognition of coded GCP, calibration panel recognition and correction, defining region of interest, extracting plot-level data [32], batch and multi-threading processing.

In this paper, we are describing a robust feature extraction platform for aerial image processing called CIAT Pheno-i, with which we validated the developed framework using cassava time series aerial images collected from two consecutive field trials (2016–2018). Since no studies have been reported on UAV based cassava high-throughput phenotyping and root yield prediction, the specific objectives of this study is (1) to develop simple and rapid aerial image analysis framework (CIAT Pheno-i) for retrieving cassava canopy variables and VIs from multispectral (MS) time series images; 2) to find promising image based canopy metrics and VIs to estimate above and below-ground biomass of cassava over different phenological stages; and (3) to develop robust ML models to predict cassava root yield using image features.

## Materials and methods

### Experimental site and trial conditions

**Fig. 1:**
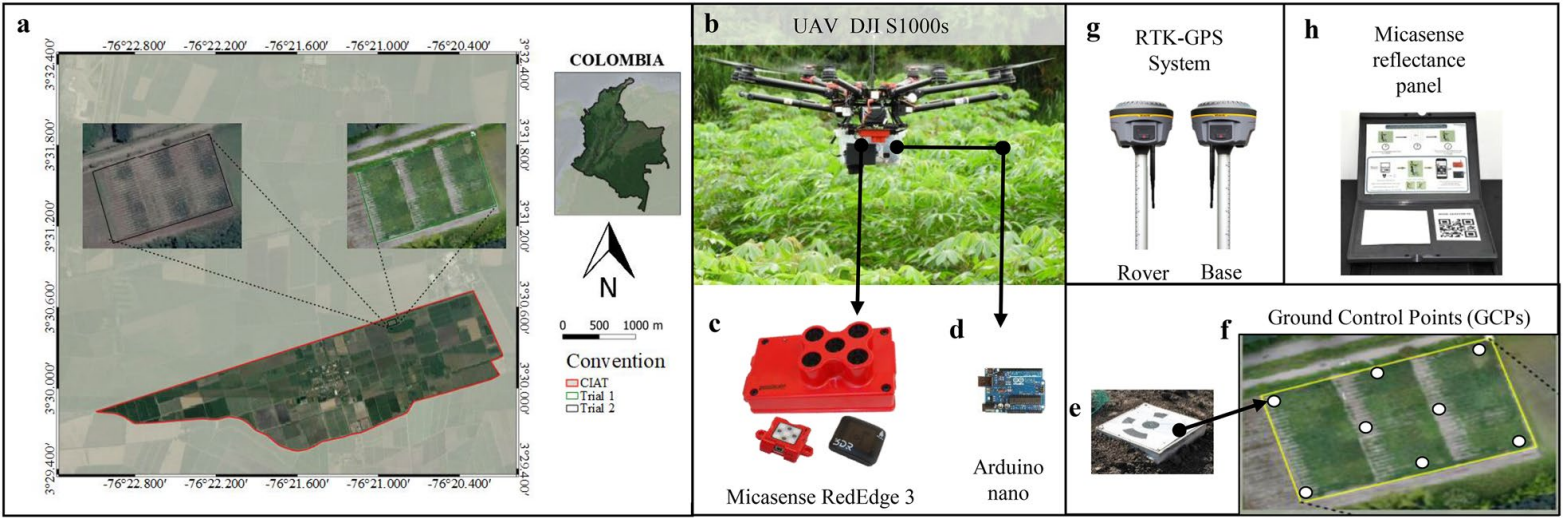
Field trial site and remote sensing platform. **a** Trial one and two were conducted at the International Center for Tropical Agriculture (CIAT). **b** Unmanned aerial vehicle (UAV), DJI S1000s. **c** Multispectral camera, Micasense RedEdge 3. **d** Arduino nano. **e** Ground Control Point (GCPs). **f** GCPs installed in trial one. **g** RTK-GPS

To validate the performance of CIAT Pheno-i, two field trials, trial one was planted on December 2016 and harvested in November 2017; trial two was planted in December 2017 and harvested in December, 2018, these trials were conducted at the International Center for Tropical Agriculture (CIAT) headquarters Valle del Cauca, Cali, Colombia at 970.67 m.a.s.l (3°30′29.21″N − 76°20′53.98″W) (Fig. 1a). Climate and experimental conditions were characterized for both trials (Table 1). For both trials, we selected four contrasting genotypes GM3893-65, CM523-7, MPER-183, and HMC-1, representing three types of canopy architecture; cylindrical, open and compact [36] and morphological and agronomic growth descriptors are listed in Additional file 1: Tables S1 and S2. The trial one was established in 0.8 hectares under a split-plot design with three replications and a total of 135 plots (3.0, long × 9.6 wide) with staggered planting (Nine planting dates from December 2016 to August 2017) (Table 1). Cuttings were planted of 1.5 m between hills and 2.4 m between rows and water management was applied by the surface irrigation system from planting to 7 months, using approximately 4000 m^3^ per hectare. The second trial was planted in 0.6 hectares with four replications per genotype and plot size of 9.6 m long and 9.6 m wide (Table 1). Cuttings were planted of 1.2 m between hills and 2.4 m between rows and water management was applied with an efficient drip irrigation system from planting to 7 months, using approximately 900 m^3^ per hectare. In both trials, stem cuttings between 20 to 25 cm were planted vertically into the soil, leaving exposed three buds. Weeds were controlled by hand weeding, brush-cutter, and applying herbicides in late cassava stages. Standard agronomic, insects and diseases management practices were followed. A recommended dose of diammonium phosphate (DAP) and potassium chloride (KCL) were applied at the rate of 35.89 and 179 kg ha^−1^, respectively.

**Table 1:** Field experimental conditions and images acquisition

Trial conditions and images acquisition	Trial one (Dec. 2016–Nov. 2017)	Trial two (Dec. 2017–Dec. 2018)
Irrigation	Surface irrigation	Drip irrigation
Soil type	Clay loam	Clay loam
Experimental design	Split plot design	Randomized complete block design
No. of replication	3	4
Average annual precipitation (mm)	1435.10	1026.50
Average annual temperature (°C)	24.00	23.33
Total solar radiation (W/m^2^)	207.8	222.39
Average annual relative humidity (%)	81.30	78.90
Image acquisition Stages	EL, LBK and DMA:	EL, EBK, LBK and DMA

The following definitions are related to cassava storage root development phases: Elongation (EL) stage is the initial growth phase of active fibrous root development. Early bulking (EBK) is the root differentiation (from fibrous and storage roots) phase, the beginning of storage root bulking and accumulation of assimilated reserves in the storage roots. Late bulking (LBK) stage is the rapid expansion and bulking of storage roots. Dry matter accumulation (DMA) stage is the starch accumulation in the storage roots

### Ground-truth measurements

Cassava agronomic traits such as leaf area index (LAI), canopy height (CH), above-ground biomass (AGB) and below-ground biomass (BGB) were acquired as ground-truth measurements. Five plants per plot were measured using LICOR LAI-2200C Plant Canopy Analyzer [37] during trial two. CH was sampled from soil level to the upper canopy at all four important phenological stages: elongation (EL), early bulking (EBK), late bulking (LBK) and dry matter accumulation (DMA) in both the trials. Each phenological stage is defined in Table 1. CH of 21 and five plants per plot were measured in trial one and two, respectively. The AGB and BGB were measured at the harvest time in both the trials using a conventional scale with the accuracy of 1 g. For AGB, three and five plants per plot were sampled in trial one and two, respectively. For BGB, 15 and 45 plants per plot were sampled in trial one and two, respectively.

### UAV platform and images acquisition

In this study, aerial multispectral (MS) time-series images were obtained using a MS camera (MicaSense RedEdge 3) mounted on a commercial UAV DJI S1000 octocopter (Fig. 1b). The MS camera has five spectral bands—Blue, Green, Red, near-infrared (NIR), and Red Edge (RE) with the wavelengths of 455–495 nm, 540–580 nm, 658–678 nm, 800–880 nm and 707–727 nm, respectively (Fig. 1c). The camera was attached to UAV by one plate with a shock absorption rubber/spring damping suspension system to protect against any vibration and to ensure better quality of the images. Six automated PhotoScan coded target detection (concentric rings) as ground control points (GCPs) were printed on a 50 × 50 cm plastic sheet (Fig. 1e) and evenly distributed within the field trial (Fig. 1f). These GCPs were georeferenced using the highly accurate RTK-GPS (Real-Time Kinematic Global Positioning System, South, Galaxy G1, China) with a horizontal accuracy of 0.25 m and a vertical accuracy of 0.5 m, which was used for geometric corrections (Fig. 1g). These GCPs were maintained until all the UAV images were acquired. The automatic fly mission was performed using DJI Ground Station Pro Application (DJI GS Pro, China). Before each image acquisition, one image was taken to the MicaSense reflectance panel for radiometric calibration (Fig. 1h). Each image acquisition was taken between 10:00 to 14:00 UTC-05:00. In order to achieve overlapping of 75% vertically and horizontally, we triggered the camera using the UAV DJI A3 flight controller and Arduino Nano as an interface configured by DJI GS app (Fig. 1d). The altitude for image acquisition was between 30 and 40 m above ground level (from 2.7 to 5.4 cm per pixel). DJI S1000, batteries, and multi-sensors weights 3 kg. DJI S1000 UAV includes a Global Navigation Satellite System (GNSS), an inertial measurement unit (IMU), barometer and compass; all these components aid in position accuracy and vertical stability of the UAV during image acquisitions. The time series UAV images captured at different phenological stages at trial one and two are listed in Table 1 and these acquired time series images used to create the orthomosaic employed structure from motion (SfM) were listed in Additional file 1: Tables S1 and S2.

### Image data processing

#### Generation of orthomosaic and digital elevation models

To ensure the reflectance quality of the orthomosaic, we followed the steps suggested by Agisoft and MicaSense RedEdge cameras (Agisoft, https://bit.ly/32swtn2). These steps include the usage of the MicaSense downwelling light sensor to fix any illumination issues caused by the weather conditions and MicaSense reflectance calibration panel. The acquired images were processed through Agisoft MetaShape Pro software (Version 1.2.2, Agisoft LLC, http://www.agisoft.com) and its Python API (Application Program Interface) generates and exports a five-band orthomosaic and digital elevation models (DEM) automatically as GeoTIFF format. Our processing workflow includes following nine main steps (1) Uploading UAV images, (2) calibration, (3) GCPs detection and geo-tagging, (4) photo alignment, (5) camera optimization (6) build dense point cloud, (7) build DEM, (8) build orthomosaic (9) export DEM and orthomosaic (Additional file 2: Figure S1). In step three, coded GCPs are automatically detected through Agisoft Metashape API (Fig. 1e).

#### Comparison of manual and automatic orthomosaic and DEM generation

In order to evaluate the efficiency of the Agisoft Metashape Python API, we generated orthomosaic and DEM using manual (M1–M8) and auto mode (A1–A8) from MS and RGB datasets. All data sets (MS and RGB) were processed using the image processing workflow listed in Additional file 2: Figure S1.

#### CIAT Pheno-i image analysis framework

The CIAT Pheno-i is a web-based application (http://pheno-i.ciat.cgiar.org/), designed to extract UAV derived vegetation indices (VIs) and canopy metrics such as canopy height (CHuav), canopy cover (CCuav) and canopy volume (CVuav) rapidly. Canopy height defines the 95th percentile pixel height of the canopy point cloud. Canopy cover is the pixel surface area covered by the canopy. Canopy volume, is the total volume under observed canopy values, which is derived as follows $\mathop \sum \nolimits_{i}^{n} CCuav_{i} *CHuav_{i}$ where $i$ is the pixel associated to the plot. CIAT Pheno-i admits MS orthomosaics and DEM as an input and visualizes them as VIs maps (Additional file 2: Figure S2). Users have the privilege to select their Region of Interest (ROI) using shapefiles and perform radiometric calibration, if necessary. Currently, eight VIs (Table 2) [17, 38–43] and three canopy metrics could be rapidly generated through Pheno-i and users can visualize real-time data captured over multiple timing points during the crop development.

**Table 2:** Summary of vegetation indices used in this study. Camera channels B: blue, G: green, R: red, RE: red-edge, and NIR: near-infrared

Vegetation index	Acronym	Formula	References
Normalized difference red-edge	NDRE	(NIR-Rededge)/(NIR + Rededge)	[38]
Normalized difference vegetation index	NDVI	(NIR-Red)(NIR + Red)	[39]
Green normalized difference vegetation index	GNDVI	(NIR-Green)/(NIR + Green)	[40]
Blue normalized difference vegetation index	BNDVI	(NIR-Blue)/(NIR + Blue)	[41]
Normalized difference vegetation index red-edge	NDREI	(Rededge-Red)/(Rededge + Red)	[42]
Normalized pigment chlorophyll index	NPCI	(Rededge-Blue)/(Rededge + Blue)	[43]
Green–red vegetation index	GRVI	(Green–Red)/(Green + Red)	[17]
Normalized green–blue difference index	NGBDI	(Green–Blue)/(Green + Blue)	[40]

#### CIAT Pheno-i software architecture

##### CIAT Pheno-i back-end

**Fig. 2:**
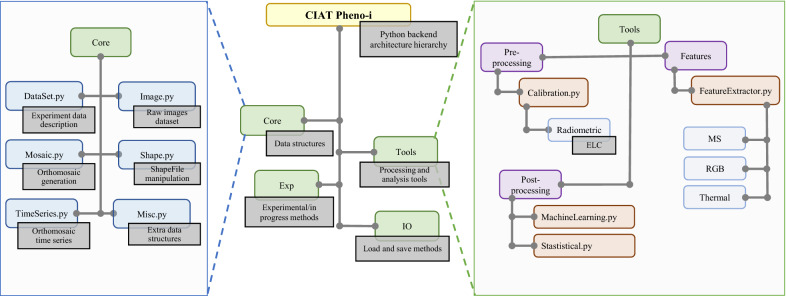
CIAT Pheno-i data processing back-end Python architecture

**Fig. 3:**
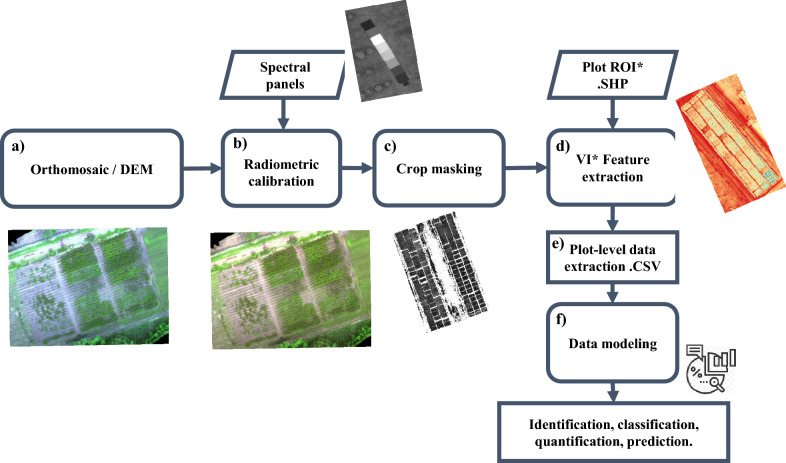
CIAT Pheno-i workflow: Applying image processing for plot level data generation and use it on identification, classification, quantification and prediction

On the top of a PostgreSQL database model, two main components constitute the Pheno-i back-end: A Python library, where the core algorithms in the pipeline were implemented (Figs. 2 and 3), and a REST (REpresentational State Transfer) API that allows the data processing through HTTP protocol. Most of the functions in the library were optimized using Numba, a python package that translates Python functions to optimized machine code, which could be executed in a parallel way on the CPU or the GPU. In addition to this, geo-spatial data manipulation, machine learning algorithms, GDAL, and Scikit-Learn were also employed. The following steps described below were coded in the CIAT Pheno-i python library:

- Step 1. Radiometric calibration: Using orthomosaics (Fig. 3a), Pheno-i back-end implements Empirical Line Calibration (ELC) process (Fig. 3b) using ground targets, allowing the user to calibrate orthomosaics after the flight. Before implanting the ELC process, the pixel digital numbers should range from 0 to 65,535 corresponding to a 16-bit standard GeoTIFF format, after applying ELC the pixel values were converted to reflectance values between 0 and 1.
- Step 2. Crop masking and Vegetation indices calculation: To segment cassava canopy, the green minus red (GMR) processing was used [44]. The binarization of GMR was determined by the Otsu method to perform clustering-based image thresholding [45], which implies the reduction of a gray level image to two-pixel values (0 and 1) and this binary image was used to select and discard the pixels associated with the soil (Fig. 3c). Using five camera channels (B: blue, G: green, R: red, RE: red-edge, and NIR: near-infrared), eight normalized vegetation indices (VIs) were intended (Table 2.)
- Step 3. Plot-level data extraction: Using the calibrated version of the orthomosaic, the boundaries of each plot ids are defined using an ESRI Shapefile format polygon. Then, shapefile was further used to select and extract the pixel values to compute statistics such as mean, variance, median, standard deviation, sum, minimum and maximum (Fig. 3e).

##### CIAT Pheno-i web

A single page app (SPA) was developed using React.js and Redux. This web application can be executed using any modern web browser (IE 11, Edge ≥ 14, Firefox ≥ 52, Chrome ≥ 49, Safari ≥ 10). The user interface follows the Material-UI v4.7.0 (https://material-ui.com/) guide design, LeafletJS v1.6.0 API (https://leafletjs.com/) was used to draw the geo-referenced orthomosaics and polygons in an OpenStreetMap (https://www.openstreetmap.org/). Additional file 2: Figures S3 and S4 shows the overall architecture and database schema implemented.

##### CIAT Pheno-i back-end performance

To evaluate the CIAT Pheno-i back-end performance, a single and multi-thread analysis were performed under server and workstation platforms over 50 different datasets. Hardware and software specifications are listed in Additional file 1: Table S3.

### Statistical analysis

To investigate the relationship between agronomic traits, VIs and canopy metrics, we conducted Pearson correlations, where the traits were calculated using a pearsonr function from Python SciPy (https://www.scipy.org/) package. Pearson’s correlation coefficients and a *P* value less than 0.05 was considered significant.

### Dataset preparation

In order to validate Pheno-i analysis, a Comma Separated Values (CSV) file with 693 characteristics was generated. Four machine-learning algorithms such as Support Vector Machine (SVM), k-Nearest Neighbours (kNN), Random Forest (RF) and Artificial Neural Networks (ANN) were evaluated to predict cassava root yield. For the pre-processing, data scaling between -1 and 1 and a Box Cox transformation were performed to achieve a normal distribution [46]. Principal component analysis (PCA) and principal component regression (PCR) [47] was applied to compare performance and reduce the model complexity providing a lower-dimensional representation of predictor variables and to avoid multi-collinearity between predictors [48–50]. To analyze the data at different growth stages, a multi-temporal VIs technique was applied [27]; this procedure increases the predictor variables from 77 to 693 per timing point accumulating the VIs value per phenological stage.

### Machine learning (ML) model development

#### ML model used

We included four ML methods in our study and are briefly described below. These ML methods were used in the regression mode.

##### Random forest

Random forest method is a non-parametric, supervised method, that can be used as both classification and regression. The heart of tree-based learners is the decision tree, wherein a series of decision rules are chained and learned. In a decision tree, every decision rule occurs at a decision node [51]. This model was proposed by Tin Kam Ho and further adapted by Leo Breiman and Adele Cutler [52].

##### Support Vector Machine

Support Vector machines [53] classify data by finding the hyperplane that maximizes the margin between the classes in the training data. A support vector machine can be represented like:$f\left( x \right) = \beta_{0} + \mathop \sum\nolimits_{{i \in S}} \alpha_{i} K\left( {x_{i} ,x_{\^i } } \right)$, where $\beta_{0}$ is the bias, $S$ is the set of all support vector observations, $\alpha$ is the parameters in the model to be learned, $\left( {x_{i} ,x_{\^i } } \right)$ are pairs of two support vector operations and K is the kernel function which compares the similarity between $x_{i} ,x_{\^i }$.

##### k Nearest Neighbors

The k-nearest Neighbors algorithm [54] is a supervised machine learning algorithm that can be used as both classification and regression problems, especially when there is little or no prior knowledge about the distribution of the data. Let $X_{i}$ be an input sample with $p$ features $\left( {x_{i1} ,x_{i2} , \ldots x_{ip} } \right)$, The Euclidean distance between the sample $x_{i}$ and $x_{l} \left( {l = 1,2, \ldots ,n} \right)$ is defined as $d\left( {X_{1} ,X_{l} } \right) = \sqrt {\left( {x_{i1} - x_{l1} } \right)^{2} + \cdots + \left( {x_{ip} - x_{lp} } \right)^{2} }$, and its neighborhood as: $R_{i} = \left\{ {X \in R^{p} :d\left( {X,X_{i} } \right) \le :d\left( {X,X_{m} } \right), \forall i \ne m} \right\}$, where $R_{i}$ represents the clusters of elements with class $m$, and $X$ the set of points belong to it. The predicted class of the new sample $x$ is set equal to the most frequent class among the k nearest training samples, which follow the rule:$d\left( {m_{i} ,X} \right) = \left\{ {d\left( {m_{i} ,X} \right)} \right\}$, where d is the distance function.

##### Multi-Layer perceptron (MLP)

A MLP is composed of multiple perceptrons or neurons, developed originally by Frank Rosenblatt [51], commonly arranged in three layers known as input layer, hidden layer (can have more than one stack of neurons) and output layer, and this kind of configuration is called Artificial Neural Network (ANN). Each input of the neurons $x_{i}$ are associated with a weight $w_{i}$ and computed as a sum as follows $z = x_{1} w_{1} + \cdots + x_{n} w_{n} = X^{T} W$, then an activation function is calculated as $f\left( z \right)$, where $f\left( z \right)$ can be any continuously differentiable function like a linear function, sigmoid or even the modern ReLU commonly used in deep learning [55].

### Assessing the quality of the model

Based on the experimental field design, a total of 609 samples were used to develop the models, three data repetitions (454 samples) were used to train and, one last repetition to test (155 samples). Regression model hyper-parameters were tuned using grid-search with ten-fold cross-validation to reduce variability and over-fitting while modeling; methods provided with scikit-learn Python package. To assess the accuracy and performance between models root median square error (RMSE), relative root mean square error (RRMSE) and the coefficient of determination (R^2^) were used. Ten-fold cross-validation was performed over SVM, RF and kNN to get optimal hyper-parameters that minimize the error and stochastic gradient descendant over ANN model to reduce the training error.

## Results and discussion

### CIAT Pheno–i: an automated image analysis framework for HTFP

**Fig. 4:**
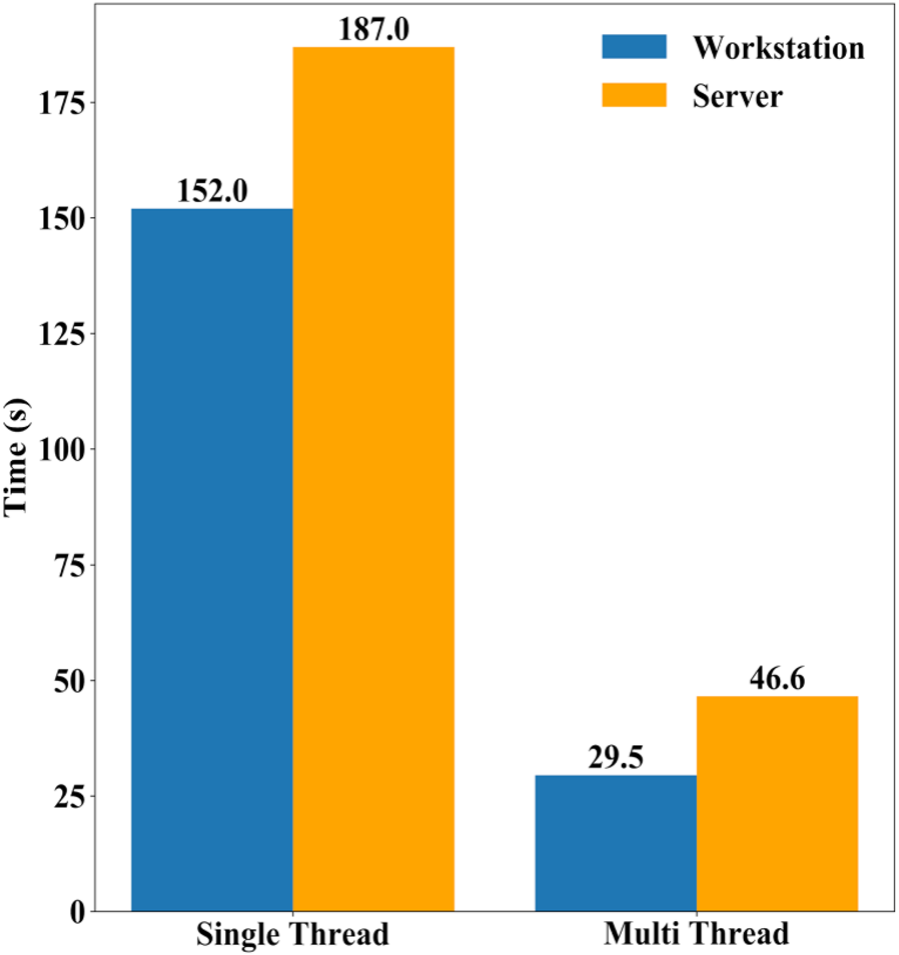
CIAT Pheno-i back-end performance. Multispectral 5 band image analysis average time of 50 runs with 3870 × 3739 pixels. Workstation processing time compared against server processing time

The increased use of UAVs in field phenotyping considerably decreased the hardware costs, however, image processing is the major challenge to the crop phenotyping scientists around the world [56]. As mentioned in the introduction, midway steps to extract information from the plot level field experiments need full automation and integration. Therefore, a need for accurate, robust, and automated analysis framework building orthomosaics and extract phenotyping information corresponding to each image of micro-plots (breeding) or large scale (precision agriculture) field experiments is necessary. Here, we are describing the Pheno-i image analysis software (Additional file 3) developed by CIAT phenomics platform (https://phenomics.ciat.cgiar.org/) and the automated orthomosaic generation pipeline. The primary criterion for any image analysis software should be cost effective, easy-to-use and rapid generation of actionable data from time-series images irrespective of experimental plot sizes. Making use of Agisoft Metashape Python API, the orthomosaic and DEM generation process was automated (Additional file 2: Figure S1), achieving a reduction in time of ~ 30%, saving ~ 1.1 h for RGB imagery and ~ 0.33 h for MS imagery (Additional file 2: Figure S5), compared to our manual processing method. CIAT Pheno-i back-end image analysis software design brings a significant improvement over any regular single thread Python implementation reducing the processing time of MS imagery processing up to 5× (Fig. 4). Aforesaid processing time was calculated using two different CPU architectures as seen in Additional file 1: Table S3. Our CIAT Pheno-i front-end software design comes with the advantage for the user to create, upload, calibrate, visualize, and analyze orthomosaics in a map-based canvas, giving a privilege to a non-programmer to analyze his own data through the internet. The image analysis report comes in CSV format with a timestamp and a reference to a quantified plot level data, in which the data can be used either to develop plant models or just to monitor the crop health status. We offered CIAT Pheno-i as a simple and easy to use solution to extract plot/plant-level information.

We validated the developed platform using proof-of-concept experiments with cassava genotypes over the two seasonal field trials to demonstrate the end-to-end application. The results obtained from the platform are described below.

### High-throughput field phenomics for aerial imaging of cassava

UAV offers very attractive alternatives such as, convenient operation, high spatial and temporal resolutions with reasonable spatial coverage [57–59], makes it possible to document the within-microplot variability in phenotyping field experiments [60, 61]. UAV, a current and an invaluable tool for crop monitoring at large scale (e.g., [27, 59, 62–65], has been proved to be useful for estimating canopy height and biomass in crops including rice [65], wheat [66] maize [30], sorghum [67] and peas [17]. However, in cassava, the UAV based high-throughput phenotyping methods need to be standardized for feasibility and accuracy in estimating various phenotyping parameters such as, biotic and abiotic stresses. So far, most studies have attempted to correlate morpho-physiological data with the productive potential (root yield) of the genotypes at the end of the crop cycle [68]. Subsequently, these pre-breeding field experiments go through long selection cycles, leading to high maintenance costs. The correlation analysis between important breeding traits at different phenological stages and UAV image derived VIs are discussed below.

### Relationship between UAV images derived features and canopy height

**Fig. 5:**
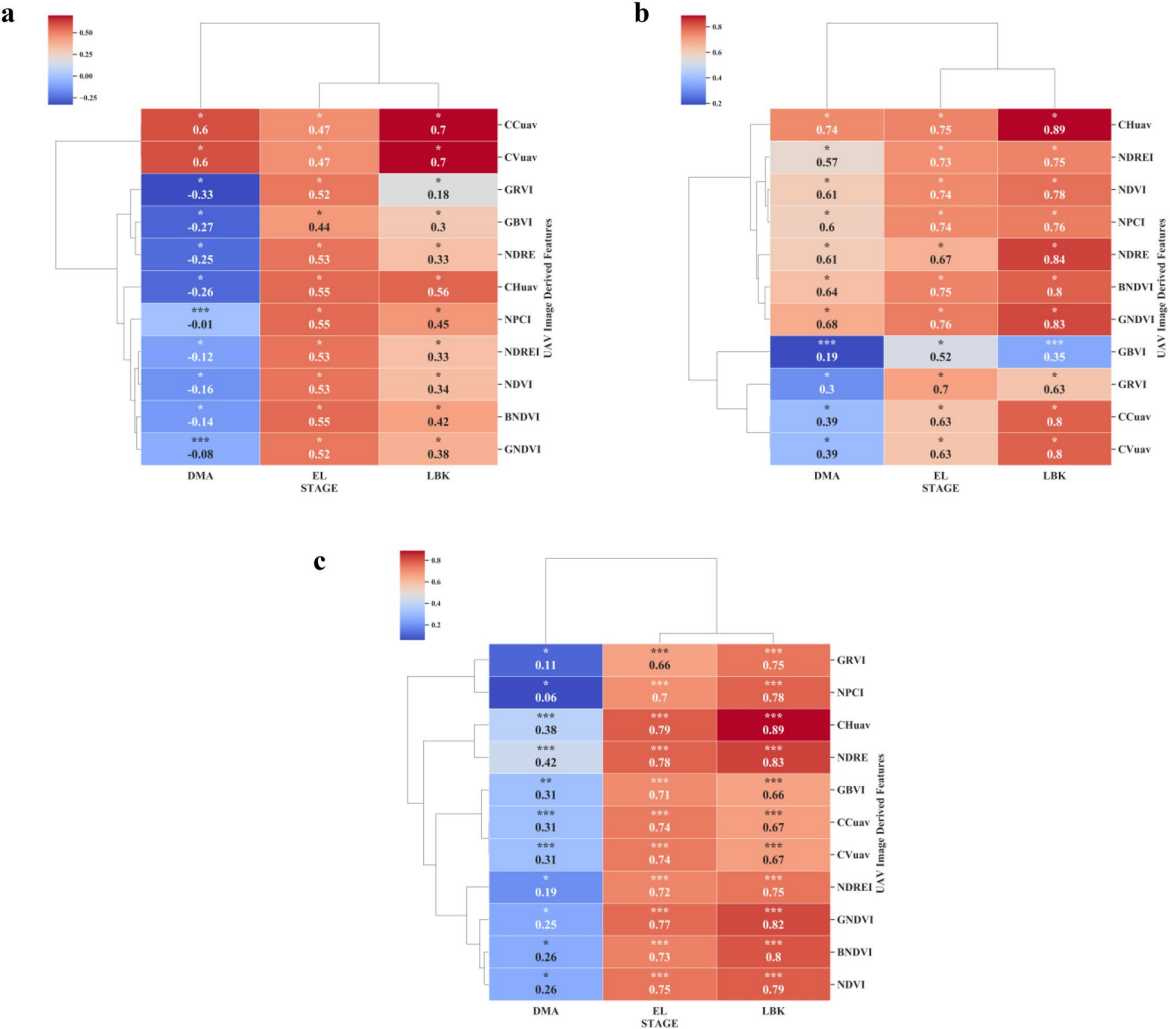
Pearson correlation analysis between remote sensing features versus shoot and root biomass at different cassava phenological stages under surface irrigation management during the trial one. **a** BGB. **b** AGB. **c** CH. P < 0.05: *, P < 0.01: **, P < 0.005: ***

**Fig. 6:**
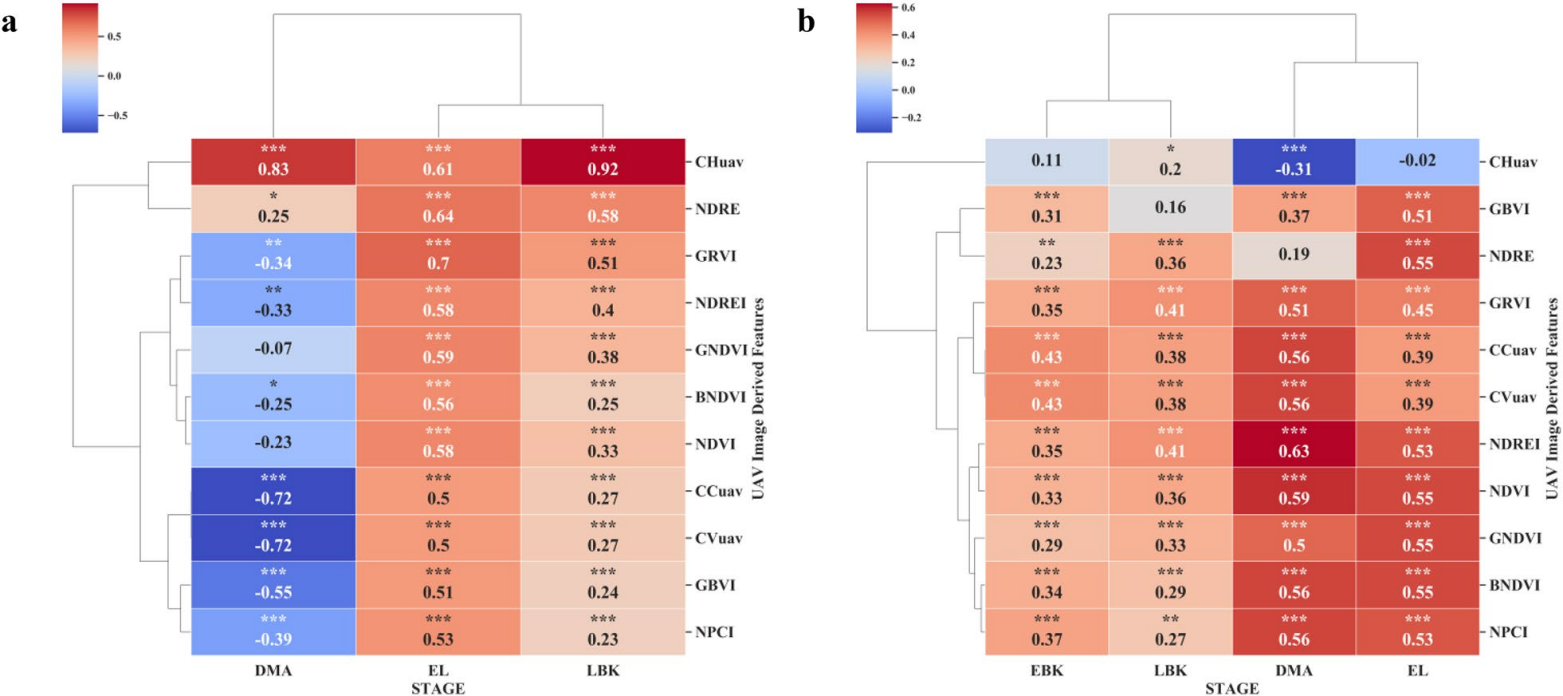
Pearson correlation analysis between remote sensing features versus canopy metric traits at different cassava phenological stages under drip irrigation management during the trial two. **a** CH. **b** LAI. P < 0.05: *, P < 0.01: **, P < 0.005: ***

**Fig. 7:**
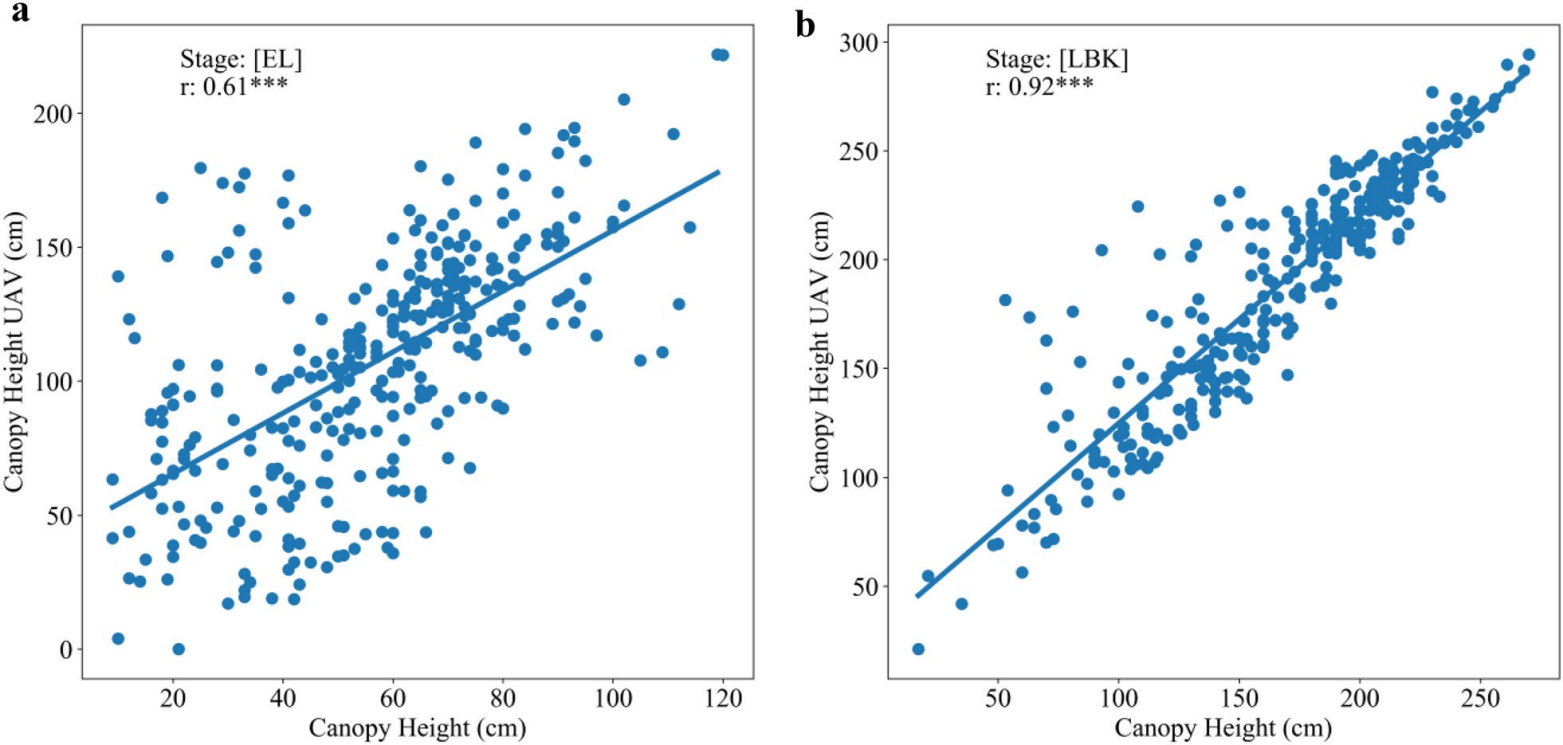
Comparison of canopy height UAV versus canopy height of cassava at EL (Elongation) and LBK (Late Bulking) during trial two

Canopy height (CH) is a key factor in cassava root yield, dry matter, leaf area, and plant architecture [69]. Collecting CH within cassava field breeding programs are labor intensive and prone to assessment error. In this study, orthomosaics and DEMs were generated using Methashape Agisoft API. Canopy metrics (CHuav, CCuav and CVuav) and VIs derived from high-resolution MS images (2.7 cm x pixel) were extracted through our CIAT Pheno-i web-based application. The pearson’s correlation analysis between UAV features (VIs, CHuav, CCuav and CVuav) and canopy height (CH) at EL and LBK stage showed that the UAV feature are positively correlated (Figs. 5c and 6a), except during the trial two, where most of the VIs showed low and negative correlations at DMA stage (Fig. 6a). This low or poor correlation is mainly due to the saturation of VIs at later stages of growth and crop lodging. Significant correlation was found at EL stage between manually estimated CH and CHuav (Fig. 7a). However, the best relationship was reached at the late bulking stage for both the trials with *r* values 0.89 and 0.92, respectively (Figs. 5c, 6a, and 7b). Similar results were found in cotton using DEMs from MS cameras [70]. In trial one, among the VIs, NDRE index showed significant relationship with CH manually with an *r* value of 0.83 at LBK stage (Fig. 5c). The CH data collected by the UAV were credible and the correlation with ground-truth measurement was very high. Therefore, UAV based CH measurements in cassava has great potential for use in studies of physiological and genetic mapping experiments.

### Relationship between UAV metrics and canopy structure related traits

**Fig. 8:**
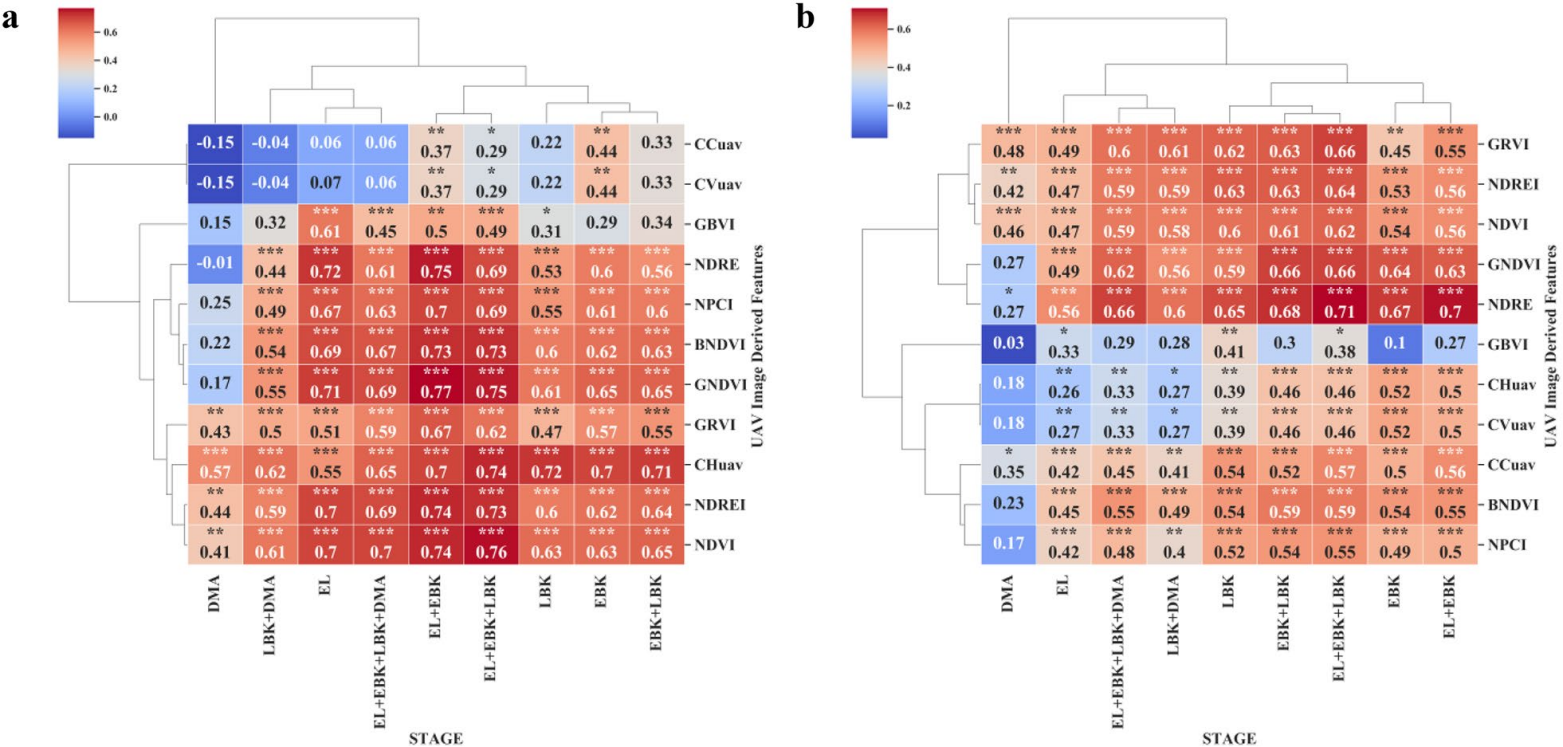
Pearson correlation analysis between remote sensing features versus shoot and root traits at different cassava phenological stages under drip irrigation management during the trial two. **a** BGB. **b** AGB. P < 0.05: *, P < 0.01: **, P < 0.005: ***

Time series measurements of canopy related traits are very useful to develop crop growth curves. Estimating AGB traits such as canopy volume is laborious, destructive and time-consuming and therefore needs an easier and convenient method [71]. In cassava, AGB can provide valuable insights into understanding the carbon assimilation mechanism and storage root development. In this paper, canopy metrics such as CCuav and CVuav across the phenological stages showed positive significant relationship with AGB. During the trial one and two, significant correlation (r = 0.80 and r = 0.54, respectively) was found between CCuav and AGB at LBK stage (Figs. 5b and 8b). A similar relationship was previously reported between dry leaf biomass and UAV derived green CC [72]. Also, at LBK stage a similar relationship (r = 0.70) was found between CVuav and BGB during the trial one (Fig. 5a). High-throughput canopy metrics tools developed from this study could provide quantitative data for novel traits that define canopy structure. Recurrent measurement offers time-series data from which we can estimate growth rates and dynamics. Such non-invasive measurements are very useful to understand genotype specific responses to environmental stresses during the growth period. Cassava canopy structure parameter data can also contribute to the development of root yield prediction models and could help cassava breeders in the selection procedure by providing early hints on the performance of novel lines.

### Correlation between LAI and UAV derived features

**Fig. 9:**
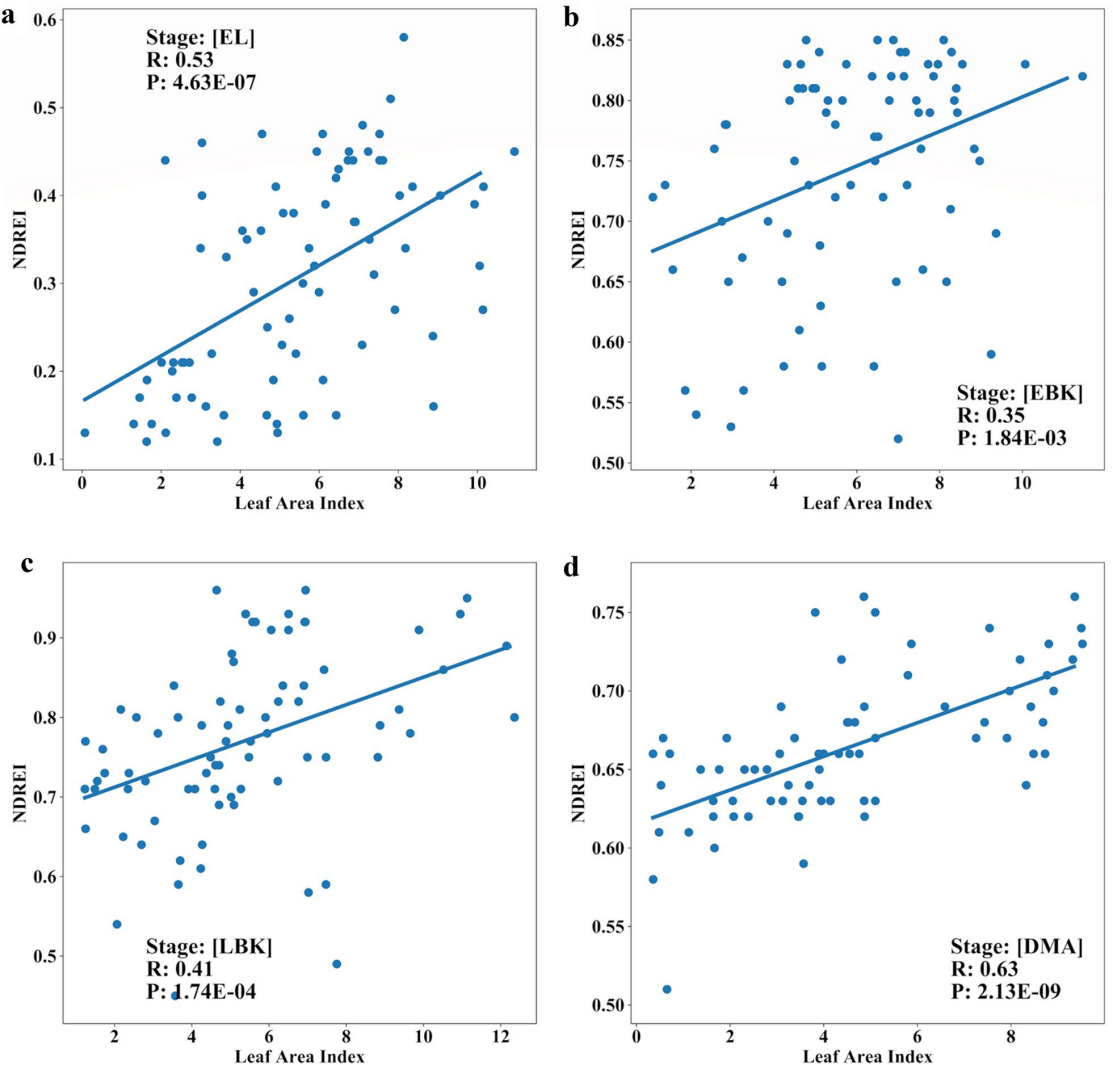
Comparison of Normalized Difference Vegetation Index Red-Edge NDREI versus Leaf Area Index (LAI) at EL (Elongation), EBK (Early Bulking), LBK (Late Bulking), and DMA (Dry Matter Accumulation) of cassava during the trial two

The leaf area index (LAI) refers to the per unit area of the one-sided leaf per unit area of ground surface. The maximum LAI in cassava ranges from 4 to 8, depending on the cultivar, the atmospheric and edaphic conditions that prevails during crop growth stages [73]. Selection for higher LAI should favor high root yield, since there is an optimum relationship between root yield and LAI [68]. Positive contribution of LAI with cassava yield has also been reported by [74], and [75] also reported significant high correlation between ground cover and LAI in grass, legume and crucifer crop. Measuring LAI is a tedious [76] and time-consuming process, and an image trait complimenting LAI can be very useful. In order to establish this relationship, in trial two, LAI was measured and the correlation analysis was performed with UAV derived canopy metrics and VIs. The results of canopy metrics (CCuav and CVuav) and VIs showed highly significant and positive correlation with LAI in all the tested phenological stages, whereas, CCuav and CVuav correlated with DMA with *r* value of 0.56 (Fig. 6b). Among the tested VIs, NDREI showed highly significant correlation with LAI at EL and DMA stage with *r* values of 0.53 and 0.63, respectively (Fig. 9a, d); whereas, the correlation decreased slightly with the bulking stages (EBK and LBK) (Fig. 9b, c). Additionally, highly significant correlations were found with LAI and NDVI at EL and DMA stages with *r* values of 0.55 and 0.59, respectively (Fig. 6b). Strong correlation between NDVI and LAI using UAV images has also been reported in different crops such as rice [65], sorghum [67]; for NDREI in bread wheat [77]. These results indicate that NDREI could explain the green leaf area during senescence.

### Relationship between UAV features and above-ground biomass

**Fig. 10:**
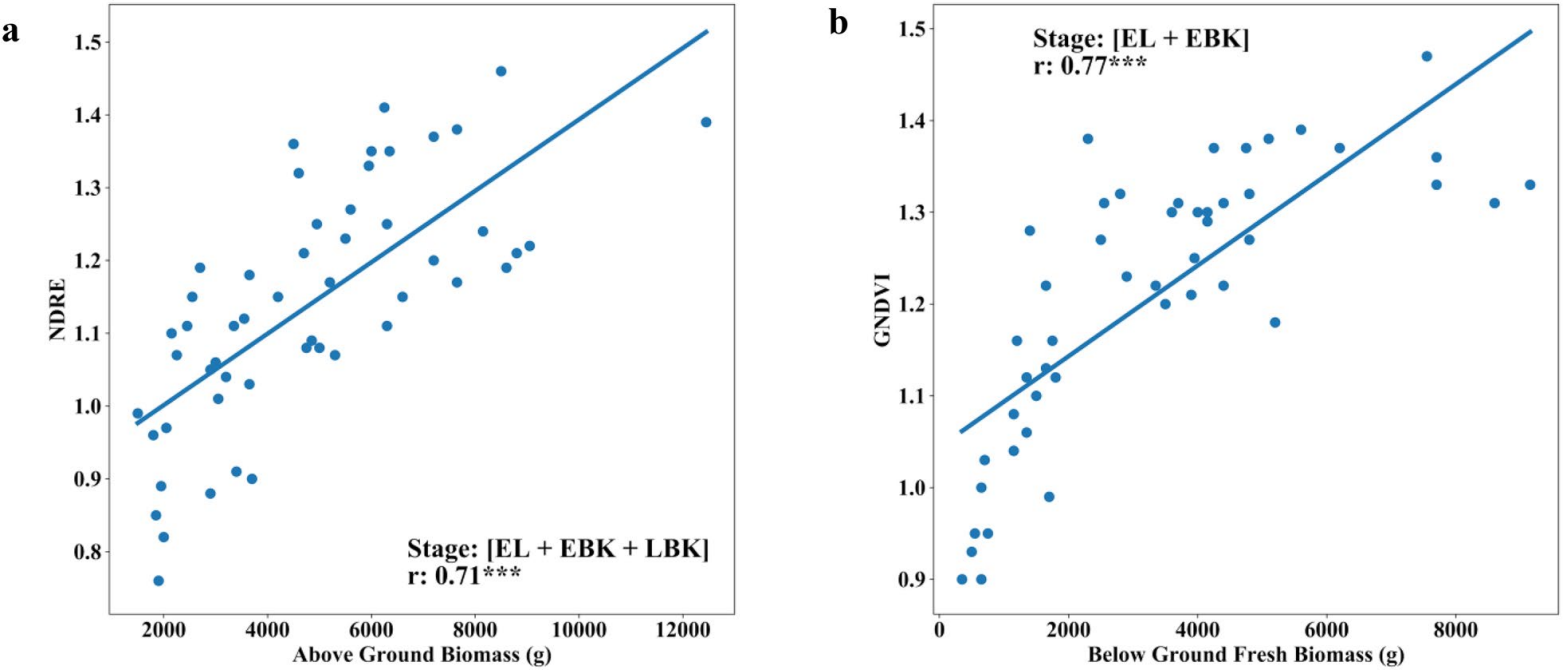
Relationship between fresh above-ground biomass (AGB) and fresh below ground biomass (BGB) of cassava with multi-temporal VIs (Normalized Difference Red-Edge, NDRE and Green Normalized Difference Vegetation Index GNDVI) during trial two. **a** Ground truth AGB versus multi-temporal NDRE index at EL (Elongation), EBK (Early Bulking), and LBK (Late Bulking stage). **b** Ground truth BGB and multi-temporal GNDVI index at EL (Elongation) and LBK (Late Bulking) stage

Breeding for early vigor, fast growing cassava genotypes is ideal to tackle several issues especially in early stages of crop management. Vigorous and early growth cultivars were less sensitive to lack of weed control than non-vigorous slow growth types. Above-ground biomass (AGB) estimation in cassava, is a most laborious and time-consuming method, requires a multi-step process: crop sacrifice from the field plot, oven dried before being weighed to assess the fresh and dry biomass of each sample. This multi-step destructive process is prone to error, from variability in the area within the plot sampled, to the potential loss of material while collecting and transporting [6]. In this present study, we estimated fresh canopy biomass in cassava using remote aerial imaging methods. Our results from both the trials revealed significant positive correlations between VIs (NDRE, NDVI, GNDVI, BNDVI, NDREI, NPCI and GRVI) and AGB, at three different phenological stages (EL, EBK and LBK). A further comparison between VIs and AGB at LBK stage, using NDRE values alone, also showed positive significant correlation in both the trials with *r* values of 0.84 and 0.65, respectively (Figs. 5b,  8b continuously differentiable function like a linear function). Across UAV derived canopy metrics at LBK stage, we found significant correlation between CCuav and AGB above *r *= 0.54 (Figs. 5b,  8b). Our results clearly indicate that EBK is one of the key phenological stages to predict AGB through remote sensing in cassava. Combining VIs at three phenological stages (EL, EBK and LBK), the trial two showed good AGB relationship with NDRE, NDVI, GNDVI, BNDVI, NDREI, NPCI and GRVI with *r* values of 0.71, 0.62, 0.66, 0.59, 0.64, 0.55, and 0.66, respectively (Figs. 8b and 10a).

### Relationship between UAV derived VIs and below-ground biomass

Measuring root biomass through non-destructive methods over different cassava varieties will help cassava breeders in the efficient selection of cultivars with favorable rooting architectures e.g. root area and harvesting [78]. Thereby, the impact of agronomic research through unique agricultural practices on root bulking can be assessed. Destructive root sampling in cassava requires sampling large populations and trials that are laborious and expensive [8]. Rapid and non-destructive process of estimating below-ground biomass (BGB) across different environments would reduce time, cost and sample size requirements in phenotypic data collection. In this study, we determine the capability of MS aerial imaging to estimate BGB. In both trials, except at DMA stage, all the tested VIs showed positive and significant correlation with fresh BGB at EL and LBK stages (Figs. 5a, 8a). Our results revealed that the later stage (DMA) of cassava crop life was least correlated, attributing the fact that at the later crop stages (i.e. when the roots are actively accumulating dry matter), cassava canopy tends to senescence.

In both the trials, NDRE, NDVI, GNDVI, BNDVI, NDREI, NPCI, GRVI indices showed significant positive correlations with fresh root biomass with *r* values ranging from 0.18 to 0.72 during the EL to LBK stage, where the highest correlation coefficient (*r *= 0.72) correspond to NDRE at the EL stage at trial two (Figs. 5a and 8a). On the other hand, canopy metrics (CCuav and CVuav) exhibited highest and stronger correlations with BGB at LBK in trial one with *r *= 0.70 and *r *= 0.70, respectively (Fig. 5a). Also, we found that the DMA stage showed poor and no significant correlation for some VIs, CHuav and CVuav metrics (Fig. 8a). In addition, the multi-temporal analysis showed improved correlations with BGB, where we observed that the combination of VIs at [EL + EBK] stages showed highly significant correlation (*r* = 0.77) for GNDVI (Figs. 8a and 10b). Generally, from 3 to 5 months after planting (MAP), intense development of the photosynthetic apparatus and aerial part of the cassava plants is observed. Consequently, a vigor in this phase causes the greatest enhancement of AGB with consequent reflection in fresh root yield [13]. The relationship between aerial imaging features and BGB obtained from this study are encouraging and it can be an add-on feature for our ongoing Ground penetrating Radar (GPR) research predicting BGB in cassava. Furthermore, all the data produced from above (UAV multispectral) and below ground sensors (GPR) could be merged using high precision Geographic Information System (GIS) to achieve more comprehensive estimation of BGB.

### Cassava root yield predictions using ML models

Accurate estimation of crop yield is essential for plant breeders. Yield is a very important harvest trait observation that involves the cumulative effect of weather and management practices throughout the entire growing cycle. [79]. Early detection and crop management associated with yield limitations can help increase productivity [4, 23, 80]. Crop yield prediction models could aid in early decision-making, optimizing the time required for field evaluation, thus reducing the resources allocated to the research programs [81]. Furthermore, the predicted yield maps could also be used to implement variable rate technology (VRT) systems in spatial databases, thereby accomplishing precise field-level inputs through the entire field [82]. Traditional cassava growth models have certain limitations, such as high input cost required to run the models, the lack of spatial information, or the actual quality of input data [13]. Remote sensing approaches can provide growers with final yield assessments and show variations across the field [79]. In remote sensing, MS imagery can describe crop development for potato tuber yield forecasting, across time and space, in a cost-effective manner [81, 82].

**Fig. 11:**
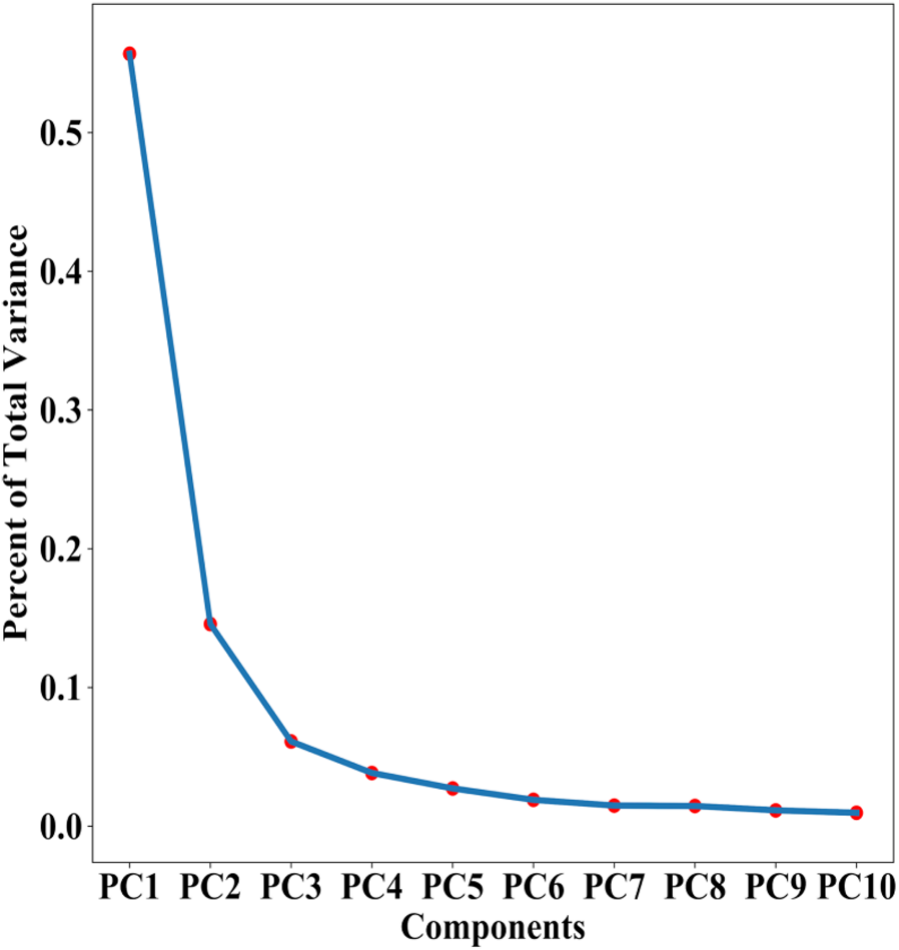
PCA scree plot of the percent of aforementioned variance during trial two

**Fig. 12:**
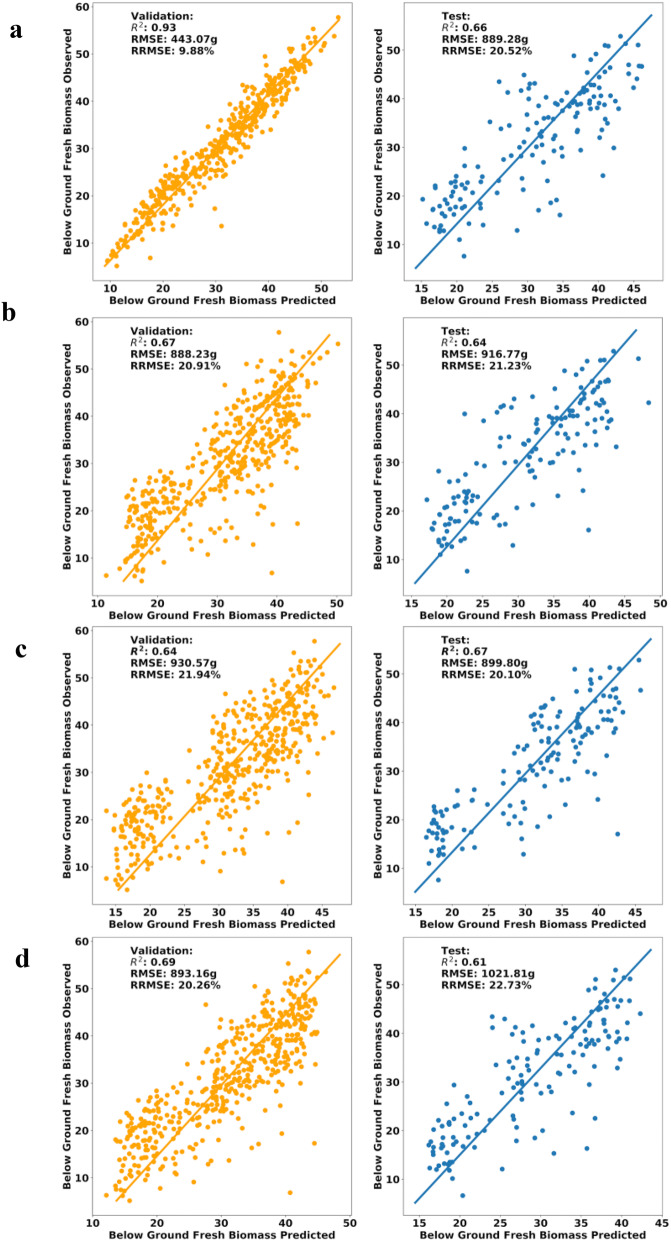
Plots based on regression methods, validation dataset on the left and test dataset on the right. **a** RF parameters (max_features:4, trees: 100). **b** SVM parameter (C:2.1, kernel:”rbf“). **c** kNN parameters (algorithm: ball_tree, K: 38, weights: uniform). **d** ANN

To our knowledge, there are no predictive models for cassava root yield using aerial imaging and ML techniques. Therefore, ML technique was explored to provide a means of early prediction of cassava root yield using MS UAV remote sensing on a field scale. A PCA and PCR analysis was used to establish, with which more than 600 predictor variables were retained to train the models. The PCA results showed that the contribution of the first 10 components explains 90% of variance (Fig. 11) and PCR after a 10 fold cross validations can achieve a R^2^ of 0.89. With PCA, the most important component was PC1, explained 55.6% of total variance (Table 3). Using the first four components provided by PCA (80% of the total variance) and PCR, SVM, RF, kNN, and ANN models were built to predict BGB using multi-temporal VIs combinations and canopy metrics (Fig. 12). Among the four developed ML models, the results showed consistent performance with small differences between PCA and PCR techniques ranging from 0 to 9% along the metrics (Table 4). PCA was performed little better than PCR in terms of RRMSE and R2 ranging from 20.51% to 22.73% and 0.61 to 0.67, respectively. In this case, the RF model gave the most well-adjusted results, with high R^2^ and lowest RMSE, indicating the importance of VIs and canopy metrics to predict BGB by MS sensors. Even though the accuracy of developed models is not very high, considering the laborious cassava phenotyping efforts, CIAT Pheno-i will still be handy for breeders to reduce their time and efforts. This model accuracy can be easily improved by adding other features such as climate, soil, and more timing points.

**Table 3:** Total variance explained by component

Component	Total variance
PC1	0.556
PC2	0.145
PC3	0.061
PC4	0.038
PC5	0.027
PC6	0.018
PC7	0.014
PC8	0.014
PC9	0.011
PC10	0.009

**Table 4:** Root yield ML Model comparison

	ML Method	PCA			PCR			PCA vs PCR Difference (%)		
		R^2^	RMSE	RRMSE	R^2^	RMSE	RRMSE	R^2^	RMSE	RRMSE
Validation	RF	0.93	443.07	9.88%	0.94	449.09	9.19%	1.07%	1.35%	7.24%
	SVM	0.67	888.23	20.91%	0.63	947.45	22.10%	6.15%	6.45%	5.53%
	kNN	0.64	930.57	21.94%	0.64	953.3	22.01%	0.00%	2.41%	0.32%
	ANN	0.69	893.16	20.26%	0.7	910.21	21.12%	1.44%	1.89%	4.16%
Test	RF	0.66	889.28	20.52%	0.64	891.86	21.12%	3.08%	0.29%	2.88%
	SVM	0.64	916.77	21.23%	0.64	874.13	21.14%	0.00%	4.76%	0.42%
	kNN	0.67	899.8	20.10%	0.67	879.09	20.20%	0.00%	2.33%	0.50%
	ANN	0.61	1021.81	22.73%	0.61	1120.04	22.61%	0.00%	9.17%	0.53%

## Conclusions and future directions

The use of UAV platforms in rapid acquisition of phenotypic information, such as key phenological stages and vegetation indices as described in this work, have great potential to be used as a selection tool in cassava breeding programs. Automated image analytical framework (CIAT Pheno-i) developed in this study showed promising results and could be applied to other crops than cassava to accelerate germplasm and varietal selection. Machine learning model to predict cassava root yield using MS UAV imagery is encouraging however further validation in diverse sets of germplasm in different environments is necessary. Furthermore, the validation of this ML models in large cassava core collection is currently under progress. In summary, UAVs equipped with MS sensors rapidly monitored canopy metrics, VIs and effectively predicted cassava root yield in a non-destructive and cost effective way. As of now, we are also exploring other ground sensor technologies such as Ground penetrating radar (GPR) to predict cassava root yield more accurately by integrating above and below-ground time series information. Through different innovative remote sensing and image technologies it is highly possible to find out the hidden secrets of below-ground information in cassava which eventually bring higher accuracy in yield prediction.

## Supplementary information

**Additional file 1: Table S1.** Cassava morphological and agronomic descriptors. **Table S2.** Phenological stage information (Months) of genotypes listed in this study. **Table S3.** List of hardware and software used in this study.

**Additional file 2: Figure S1.** Agisoft Metashape automated orthomosaic building pipeline. **Figure S2.** CIAT Pheno-i Front-end overview. **Figure S3**. Pheno-i image analysis platform design. Back-end: Developed in Python 3 and Flask as a Web Service. Front-end: Developed using React as a single page app, it implements leaflet.js to render maps. **Figure S4.** Schematic representation of database implemented in CIAT Pheno-i. **Figure S5.** Comparison of time series data between manual and automatic orthomosaic generation with MS and RGB sensors. (**a)** Multispectral trial in manual mode. (**b)** Multispectral trial in auto mode. (**c)** RGB trial in manual mode. (**d)** RGB trial in auto mode. *M1-M8 Manual orthomosaics. *A1-A8 Automatic orthomosaics.

**Additional file 3.** Video of Pheno-i Image Analysis Platform (https://youtu.be/hnq_ydC1-rw).

## Data Availability

The data used in this study is available from the corresponding author on reasonable request.

## Abbreviations

AGB: Above-ground biomass
ANN: Artificial neural networks
API: Application program interface
BGB: Below-ground biomass
BNDVI: Blue normalized difference vegetation Index
CCuav: Canopy cover estimate from UAV
CH: Canopy height
CHuav: Canopy height estimate from UAV
CIAT Pheno-i: Automated image analytical framework
CPU: Central processing unit
CSV: Comma separated values
DEM: Digital elevation model
DMA: Dry matter accumulation
EBK: Early bulking
EL: Elongation
ELC: Empirical line calibration
GNDVI: Green normalized difference vegetation index
GCPs: Ground control points
GRVI: Green-Red vegetation index
GNSS: Global navigation satellite system
GUI: Graphical user interface
GIS: Geographic information system
GMR: Green minus red
GPR: Ground penetrating radar
GDAL: Geospatial data abstraction library
HTFP: High-throughput field phenotyping
KNN: k-Nearest neighbors
LAI: Leaf area index
LBK: Late bulking
ML: Machine learning
MLP: Multi-Layer perceptron
MS: Multispectral
NIR: Near-infrared
NDRE: Normalized difference Red-Edge
NDVI: Normalized difference vegetation index
NDREI: Normalized difference Vegetation Index Red-Edge
NGBDI: Normalized Green–Blue Difference Index
NPCI: Normalized pigment chlorophyll Index
CVuav: Canopy volume estimate from UAV
RTK-GPS: Real-time kinematic global positioning system
RF: Random forest
REST: Representational state transfer
IMU: Inertial measurement unit
RGB: Red, green, blue
RMSE: Root median square error
PCA: Principal component analysis
PCR: Principal component regression
RRMSE: Relative root median square error
SPA: Single page app
SVM: Support Vector Machine
UAV: Unmanned aerial vehicles
VIs: Vegetation indices
VRT: Variable rate technology
