# Supplementary material for: Machine learning for high-throughput field phenotyping and image processing provides insight into the association of above and below-ground traits in cassava (Manihot esculenta Crantz)
Source: Plant Methods. 2020 Jun 14;16:87. doi: 10.1186/s13007-020-00625-1 (PMC7296968; doi:10.1186/s13007-020-00625-1)
Supplement: Supplementary file 1 — Additional file 1: Table S1. Cassava morphological and agronomic descriptors. Table S2. Phenological stage information (Months) of genotypes listed in this study. Table S3. List of hardware and software used in this study. [file 13007_2020_625_MOESM1_ESM.docx]

**Table S1.** Cassava morphological and agronomic descriptors.

| **Genotype** | **Parents** | **Type** | **Root Architecture** | **Canopy Architecture** | **Branching Habit** | **Dry matter (%)** | **Yield (ton/ha)** |
| --- | --- | --- | --- | --- | --- | --- | --- |
| HMC-1 | CMC-9 | Commercial | Pedunculate | Open | Dichotomous | 31.6 | 35.3 |
| MPER-183 | Pure | Commercial | Mixed | Compact | Trichotomous | 34.7 | 48.1 |
| CM523-7 | CMC-86 x Guajiba | Commercial | Pedunculate | Compact | Trichotomous | 35.5 | 24.0 |
| GM3893-65 | AM816-1 x AM206-5 | Breeding line | Sessile | Cylindrical | Erect | 33 | 16.2 |

**Table S2.** Phenological stage information (Months) of genotypes listed in this study.

| **Genotype** | **Elongation (EL)** | **Early Bulking (EBK)** | **Late Bulking (LBK)** | **Dry matter accumulation (DMA)** |
| --- | --- | --- | --- | --- |
| HMC-1 | 3 to 4 | 5 to 7 | 8 to 9 | 10 to 11 |
| MPER-183 | 3 to 4 | 5 to 8 | 9 | 10 to 11 |
| CM523-7 | 3 to 4 | 5 to 7 | 8 to 9 | 10 to 11 |
| GM3893-65 | 3 to 5 | 6 to 7 | 8 to 9 | 10 to 11 |

**Table S3.** Lists of hardware and software used in this study.

| **Platform** | **Hardware and software** | **Specifications** |
| --- | --- | --- |
| **Server** | Memory | 128 GB |
|  | Processor | Intel Xeon E5-2667 v4 @ 3.20 GHz x16 |
|  | GPU | NVIDIA Tesla M60 |
|  | Operating system | Windows Server 2016 x64 |
|  | Programming language | Python |
| **Workstation** | Memory | 32 GB |
|  | Processor | Intel Core i7-8750H @ 2.2 GHz x12 |
|  | GPU | NVIDIA GTX 1050 TI |
|  | Operating system | Windows 10 Pro |
|  | Programming language | Python |
