# Supplementary material for: Machine learning for high-throughput field phenotyping and image processing provides insight into the association of above and below-ground traits in cassava (Manihot esculenta Crantz)
Source: Plant Methods. 2020 Jun 14;16:87. doi: 10.1186/s13007-020-00625-1 (PMC7296968; doi:10.1186/s13007-020-00625-1)
Supplement: Supplementary file 2 — Additional file 2: Figure S1. Agisoft Metashape automated orthomosaic building pipeline. Figure S2. CIAT Pheno-i Front-end overview. Figure S3. Pheno-i image analysis platform design. Back-end: Developed in Python 3 and Flask as a Web Service. Front-end: Developed using React as a single page app, it implements leaflet.js to render maps. Figure S4. Schematic representation of database implemented in CIAT Pheno-i. Figure S5. Comparison of time series data between manual and automatic orthomosaic generation with MS and RGB sensors. (a) Multispectral trial in manual mode. (b) Multispectral trial in auto mode. (c) RGB trial in manual mode. (d) RGB trial in auto mode. *M1-M8 Manual orthomosaics. *A1-A8 Automatic orthomosaics. [file 13007_2020_625_MOESM2_ESM.pptx]

## Slide 1
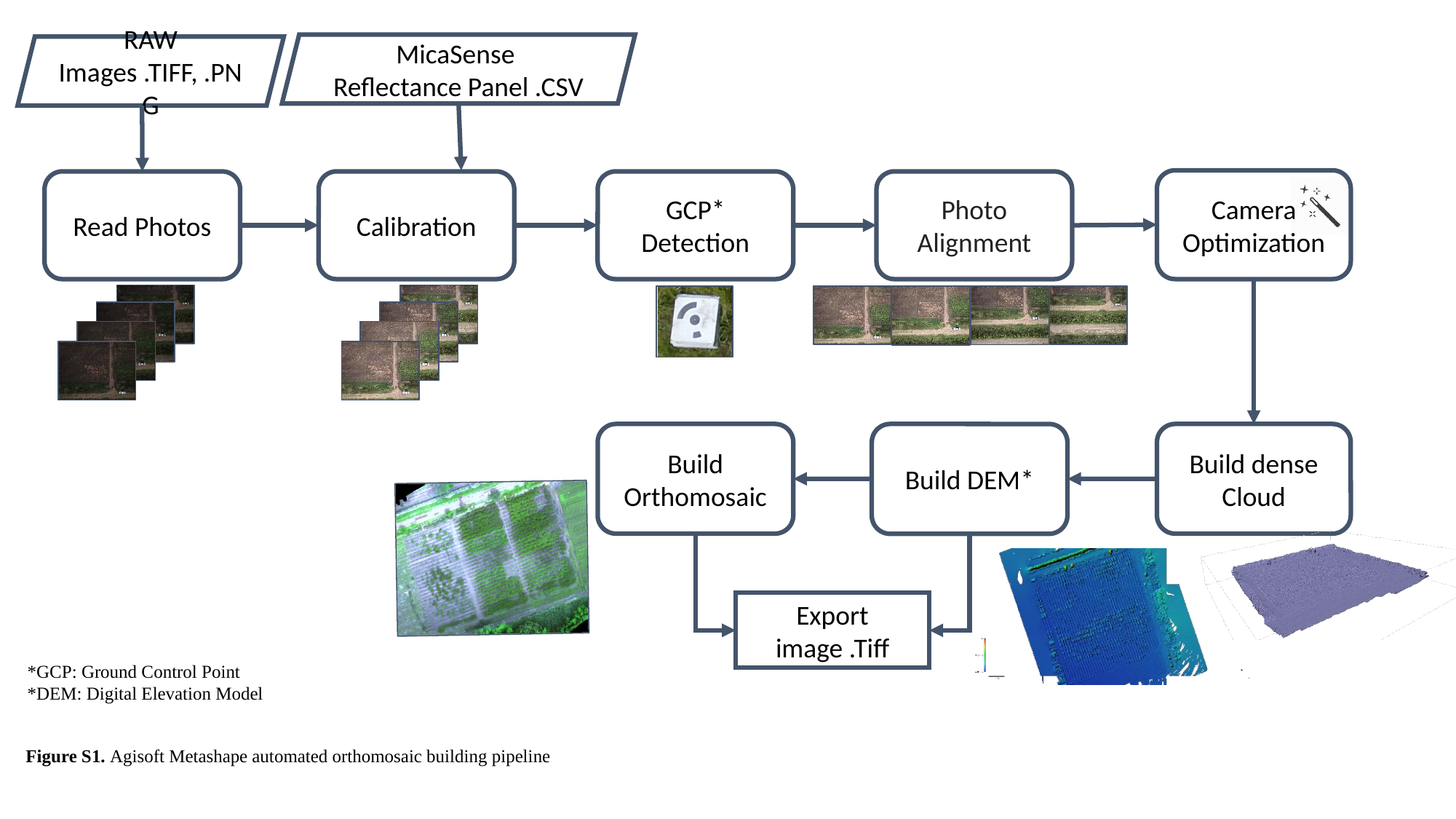

MicaSense
Reflectance Panel .CSV
RAW Images .TIFF, .PNG
Camera
Optimization
Read Photos
Calibration
GCP* Detection
Photo Alignment
Build Orthomosaic
Build DEM*
Build dense Cloud
Export image .Tiff
*GCP: Ground Control Point
*DEM: Digital Elevation Model
Figure S1. Agisoft Metashape automated orthomosaic building pipeline

## Slide 2
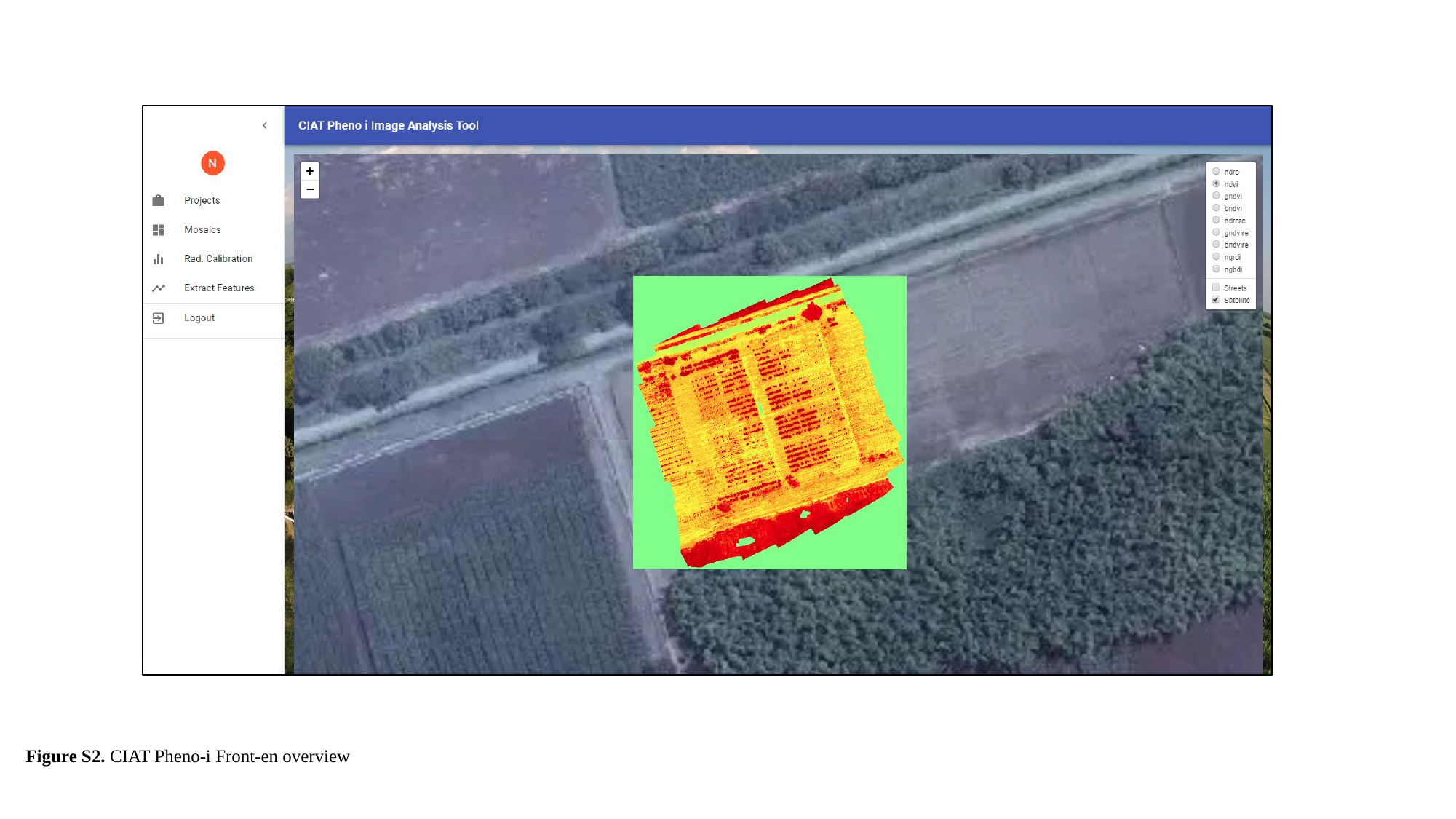

Figure S2. CIAT Pheno-i Front-en overview

## Slide 3
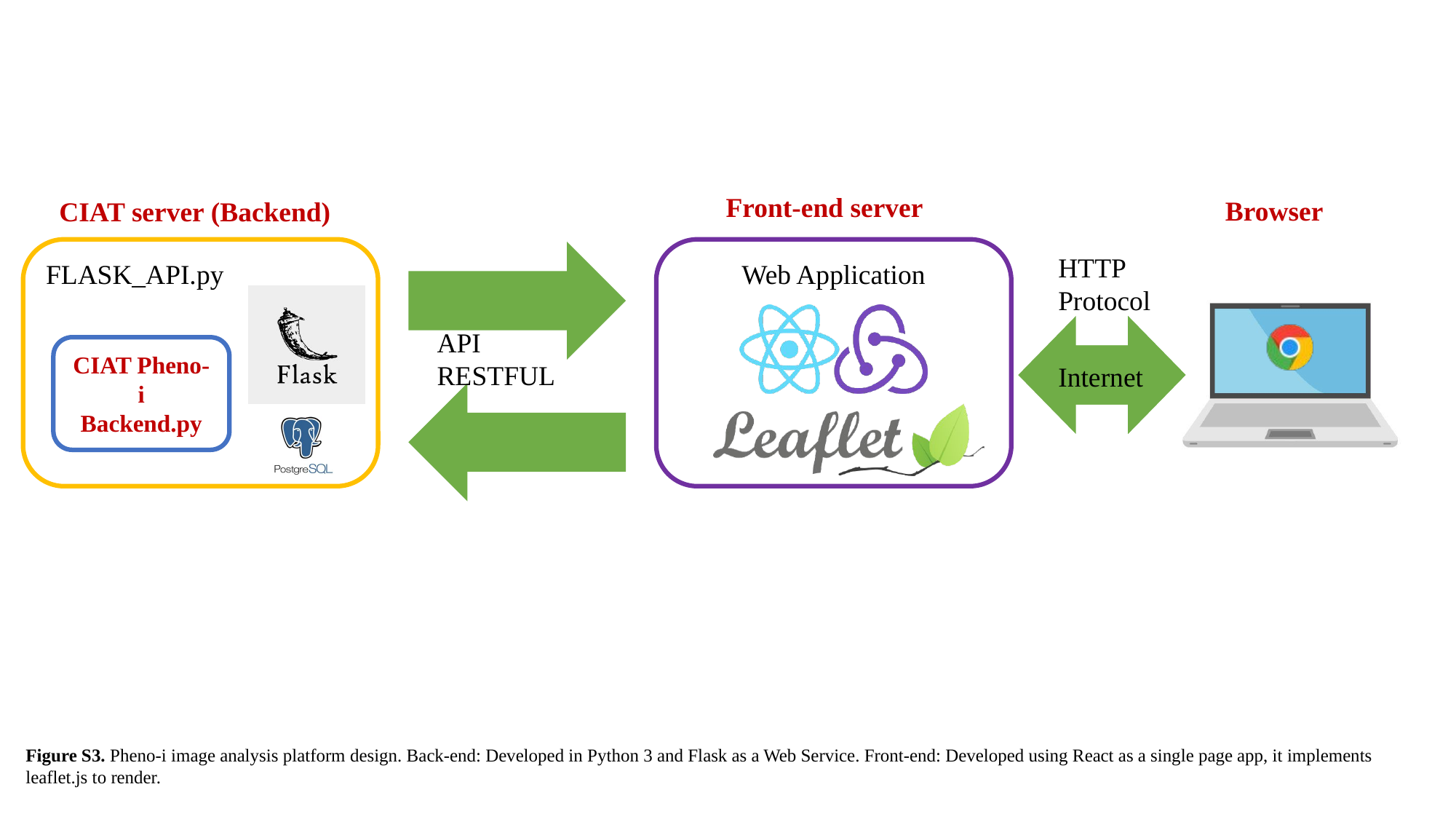

Front-end server
 Browser
 CIAT server (Backend)
FLASK_API.py
CIAT Pheno-i
Backend.py
Web Application
API RESTFUL
HTTP Protocol
Internet
Figure S3. Pheno-i image analysis platform design. Back-end: Developed in Python 3 and Flask as a Web Service. Front-end: Developed using React as a single page app, it implements leaflet.js to render.

## Slide 4
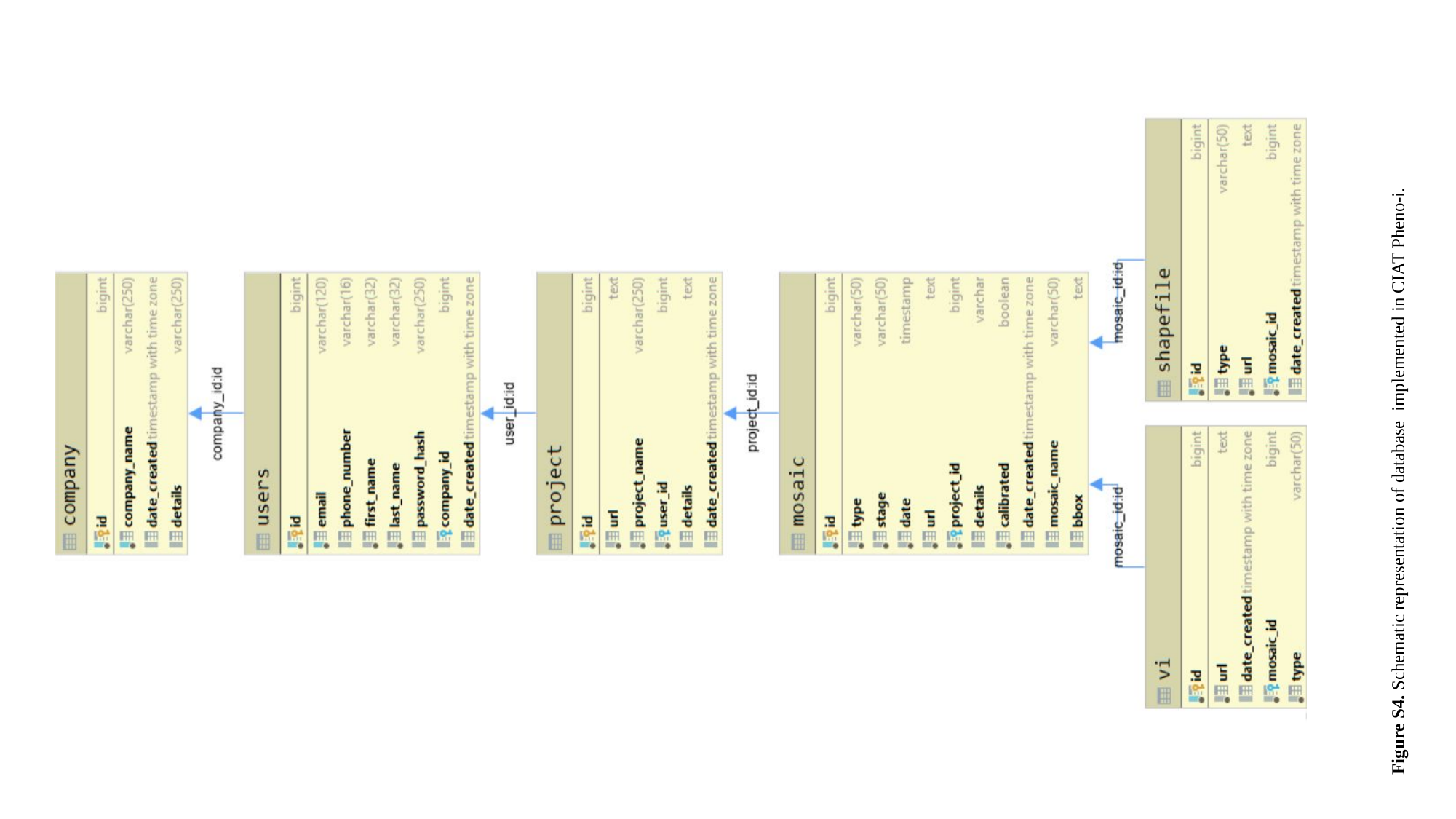

Figure S4. Schematic representation of database implemented in CIAT Pheno-i.

## Slide 5
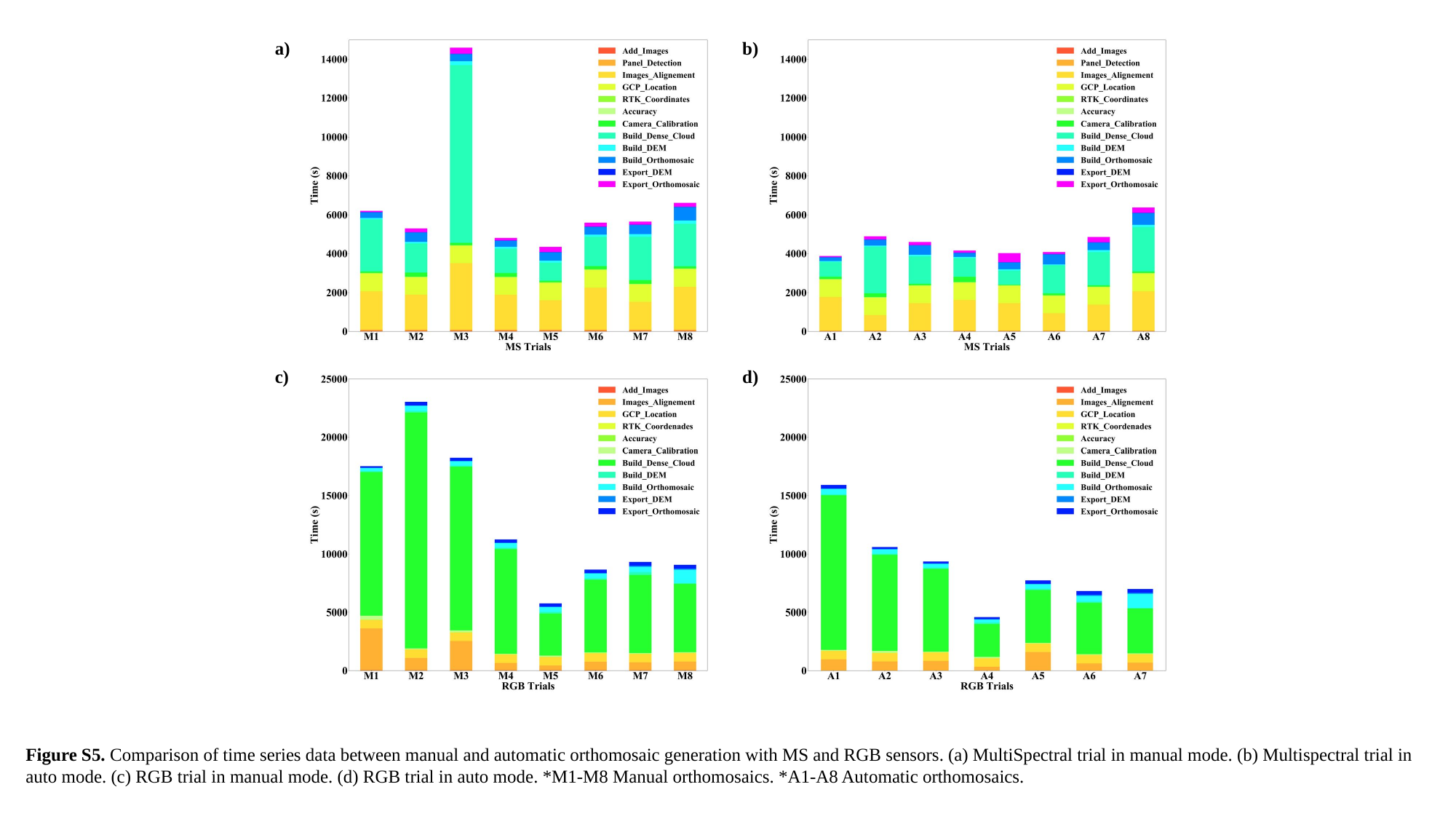

a)
b)
d)
c)
Figure S5. Comparison of time series data between manual and automatic orthomosaic generation with MS and RGB sensors. (a) MultiSpectral trial in manual mode. (b) Multispectral trial in auto mode. (c) RGB trial in manual mode. (d) RGB trial in auto mode. *M1-M8 Manual orthomosaics. *A1-A8 Automatic orthomosaics.
